# Development of a new model for estimating maternal mortality ratio at national and sub-national levels and its application for describing sub-national variations of maternal death in Ethiopia

**DOI:** 10.1371/journal.pone.0201990

**Published:** 2018-08-06

**Authors:** Samson Gebremedhin

**Affiliations:** School of Public Health, Hawassa University, Hawassa, Ethiopia; London School of Economics and Political Science, UNITED KINGDOM

## Abstract

**Background:**

In developing countries lacking functional vital registration system, statistical models are being increasingly used for estimating maternal mortality ratio (MMR). Yet, most of the models have limited applicability at sub-country level. This paper introduces a new model for estimating MMR at national and sub-national levels based on maternal health-related indicators. Further, it applies the model for explaining sub-national variations of MMR in Ethiopia.

**Methods:**

Country level data on MMR and other nine potential predictors of maternal death were extracted from 248 national Demographic and Health Surveys and other related surveys conducted in 80 low- and middle-income countries since 1990. Additional data were obtained from the World Bank and the World Health Organization databases. The potential model predictors were: contraceptive prevalence rate (CPR); utilizations of antenatal care (ANC), health institution delivery (HID), Caesarian section (CS) and postnatal care (PNC); and prevalence of maternal anemia, thinness (Body Mass Index (BMI < 18.5 kg/m^2^), short stature (height less than 145 cms) and HIV. Stepwise Generalized Estimating Equation (GEE) with Negative Log-binomial link was employed to model MMR as a function of the covariates.

**Results:**

The ultimate model comprised six significant predictors and the equation is provided as: *Ln (MMR) = 6*.*464–0*.*013(CPR)– 0*.*006(HID)– 0*.*003(PNC)– 0*.*027(CS rate) + 0*.*060(HIV prevalence) + 0*.*011 (thinness prevalence)*. The variation explained by the model was 54.3%) and the mean (±SD) relative standard error (8.6±2.6%) suggested the model has a reasonable precision. Application of the model to describe sub-national variation of maternal mortality in Ethiopia indicated, in 2016 the highest MMRs per 100,000 live births were in Somali (805) and Afar (795) regions. Between 2000 and 2016, all the regions of Ethiopia have significantly reduced MMRs; yet the rate of decline was lower in the aforementioned two regions. Oromiya region contributed for 46% of all maternal deaths in Ethiopia.

**Conclusion:**

In developing countries lacking dependable maternal mortality data; the model can be used to estimate national and sub-national MMR with reasonable accuracy and precision.

## Background

Maternal mortality refers to death that occurs to a woman during pregnancy, childbirth or postpartum period from any cause related to or aggravated by the pregnancy or its management but not from accidental or incidental causes [1]. Major direct causes of maternal death are maternal hemorrhage, unsafe abortion, maternal hypertensive disorders, sepsis and obstructed labor; whereas, important indirect causes include anemia, human immunodeficiency virus (HIV), malaria and heart diseases [2,3].

In the last three decades the world has made remarkable gains in reducing maternal deaths. Between 1990 and 2015, the estimated numbers of deaths were reduced by 43% from 532,000 to 303,000. In the same period, maternal mortality ratio (MMR)–the number of maternal deaths per 100,000 live births (LB)–declined from 385 to 216. Across different regions of the World Health Organization (WOH)) the observed rate of decline varied from 43% in the Western Asia to 72% in Eastern Asia [3,4].

However, the recent progresses falls short of the Millennium Development Goal (MDG) -5 target of reducing MMR by three quarters between 1990 and 2015. Among the WHO member state countries only ten achieved the MDG target. According to a recent estimate, globally each day about 800 maternal deaths occur from largely preventable causes. About 99% of all the deaths happen in the developing countries and the probability of women dying from complications of pregnancy and child birth is nearly 20 times higher in the developing than in the developed world. With the rate of progress observed in the last three decades, the Sustainable Development Goal (SDG) -3 target for all countries to lower MMR to less than 70 by 2030 will remain unattainable [3,4].

In low- and middle-income counties one of the key challenges for assessing progress towards maternal mortality goals is lack of reliable and accurate data [4]. Most of the developing countries do not have functional civil registration system and the situation is unlikely to change very soon. Currently many counties are using periodic national-level surveys like the Demographic and Health Survey (DHS) and the Multiple Indicator Cluster Survey (MICS) as the primary source of maternal mortality data [5]. Countries like Bangladesh, Ghana, Egypt and Morocco also conduct specialized maternal mortality surveys [6–9]. Estimation of maternal mortality through surveys is also an intricate undertaking because; from statistical perspectives maternal death is a rare event hence it can’t be accurately determined in the usual surveys [10]. Thus, surveys have to include unmanageably large samples and measure maternal mortality over a long reference period [10].

In order to fill the information gap, recently model-based estimation of MMR is being increasingly employed [3,4,11–13]. The United Nations Maternal Mortality Estimation Inter-Agency Group (MMEIG) estimates maternal mortality at national level using a regression model based on gross domestic product (GDP), general fertility rate (GFR) and skilled attendance at birth [4]. Recently a Bayesian approach to the estimation of maternal mortality (BMat model) using the same set of covariates was introduced [4,11]. Further, the Global Burden of Diseases (GBD) initiative has provided national estimates using the cause-of-death ensemble modeling [3]. A regression-based model has also been used to describe maternal mortality in Bangladesh [13].

Model-based estimates by the MMEIG has so far availed reliable national estimates of maternal mortality for most of the countries [4]. Yet, it has not been employed at sub-national level possibly due to a variety of reasons including lack of information about GDP and GFR at sub-country level. Furthermore, the fact that GDP is an important component of the model might have made it less appealing to health decision makers because improving the economic status of the country is not considered as the primary responsibility of the health system.

This paper describes the development and application of a new model for estimating MMR at national and sub-national levels exclusively based on health indicators including contraceptive prevalence rate (CPR), utilization of maternal health services, access to emergency obstetric care, women’s nutritional status and prevalence of human immunodeficiency virus (HIV). It also applies the model for explaining sub-national variations of maternal mortality in Ethiopia.

## Methods

### Study design

Country level data on MMR and nine potential predictors of MMR were extracted from national Demographic and Health Surveys (DHS) and other surveys supported by the DHS program. The study was limited to DHS supported studies because the design, approach of measurement and classification of variables of interest were roughly consistent.

### The DHS approach for estimation of maternal mortality ratio

Demographic and Health Surveys typically determine Pregnancy Related Mortality Ratio (PRMR) based on the direct sisterhood method and interpret it as a proxy of MMR [14]. Women survey respondents are asked to list all their siblings, and for each sibling information about their survival status is explored. In the case of female siblings who have died at age 12 or older, the interviewer inquires about years since death and, whether or not the sister died during pregnancy, childbirth, or within two months following delivery. In the recent surveys, additional questions are asked to determine if the death was from accidental causes or not. Ultimately PRMR over a typical reference period of 5 to7 years is determined. However, rarely PRMR is measured over a longer reference period [10,15].

### Inclusion criteria and selection of surveys

**Fig 1:**
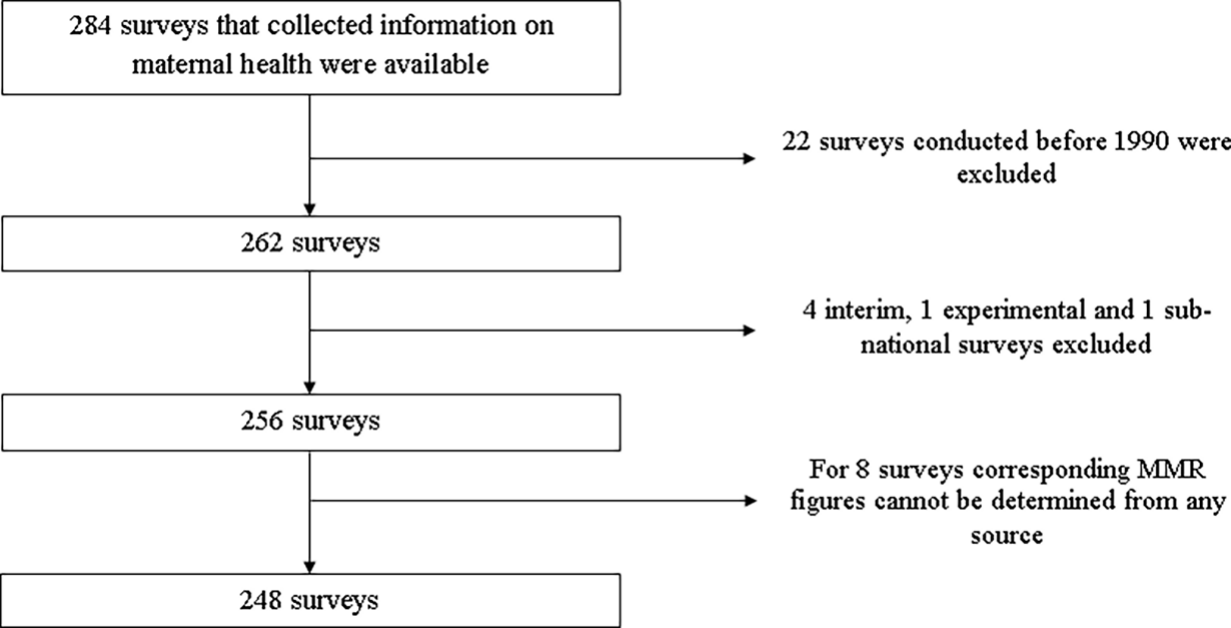
Flow chart of the study.

The analysis was conducted based on standard or continuous DHSs and other DHS sponsor surveys that collected maternal health related information. The other surveys were Maternal Health Surveys (MHS), National Family and Health Surveys (NFHS) and a specialized maternal mortality survey. DHS supported AIDS Indicator Surveys (AIS), Malaria Indicator Surveys (AIS) and Service Provision Assessment (SPA) surveys were not considered eligible for the study. The surveys were selected irrespective of whether they collected maternal mortality data or not. The survey reports were also enrolled with no geographical or language restrictions. The analysis was ultimately made based on 248 surveys conducted between 1990 and 2018. Studies carried out before 1990 were excluded because they often have missing information for most of the variables of interest. The following flow chart describes the procedure of identification of the surveys (Fig 1).

### Conceptual framework and variables of the study

**Fig 2:**
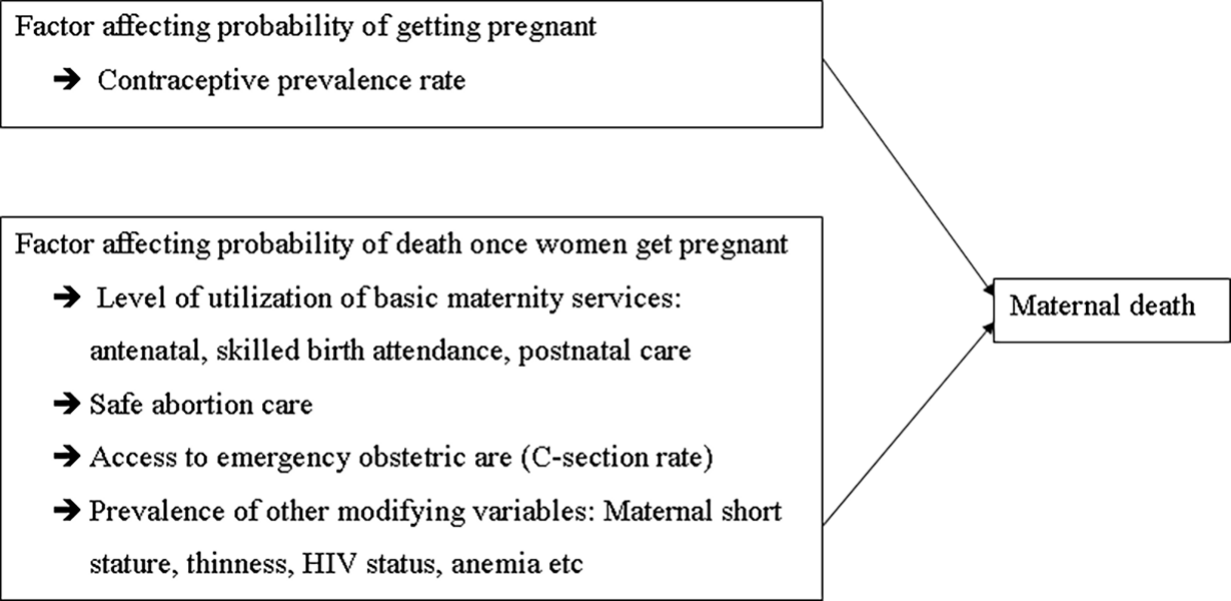
Conceptual framework of the study.

The potential predictors of maternal death were selected using the following conceptual framework. The framework assumes the risk of maternal death is affected by two factors: (*i*) probability a woman to get pregnant and (*ii*) the risk of developing obstetric complications once the woman gets pregnant and factors that improve the outcomes for women with complications. The framework assumes the probability of death after getting pregnant is modified by utilization of basic materiality services, access to safe abortion and emergency obstetric care and other effect modifiers including maternal anemia, undernutrition and HIV status [2,16] (Fig 2).

The conceptual framework was developed based on the model developed by McCarthy and Maine in 2002 [16]. The McCarthy–Maine model assumes that maternal mortality is determined by three intermediate outcomes: (i) factors like utilization of family planning service that affect the likelihood that a woman will get pregnant; (ii) factors that affect the probability that the woman would experience serious obstetric complications; and (iii) factors that may improve the outcome once the woman faces complications including access to and utilization of maternity services [16].

Based on the conceptual framework nine variables were considered as potential predictors of MMR. The variables were: modern contraceptive prevalence rate (CPR) among married women of reproductive age; proportion of mothers who had 4 or more antenatal (ANC) visits, health institution delivery and postnatal care (PNC) in the first two days after delivery for the recent birth in the last 5 years; proportion of women who had Caesarian Section (C-section) for recent birth in the last 5 years; prevalence of anemia during pregnancy; and prevalence of HIV, thinness (Body Mass Index (BMI < 18.5 kg/m^2^) and short stature (height less than 145 cms) among women of reproductive age. The analysis did not include safe abortion care—related variable as such information is available neither in DHS nor in other international databases.

### Extraction of information from surveys and management of missing data

All the 248 survey reports were downloaded from the Measure DHS website and carefully reviewed by the author. Information including basic characteristics of the surveys (country, year and type of survey), the reported MMR when available, and the level of the nine predictors were extracted into an excel spreadsheet. The dataset analyzed is provided as a supporting file with this manuscript (S1 Appendix).

Information about modern CPR, proportion of mothers who had four or more ANC visits, delivery in health institutions and percentage who had CS for the recent birth were all consistently available across the surveys so data missingness was not a problem. However, for the other variables, missing values were encountered.

MMRs were reported by 152 of the surveys. Accordingly, for the remaining surveys it was estimated using various approaches including: linear interpolation or extrapolation based on the available DHS surveys for the same country, reports of other national surveys or civil registration systems and estimates of the WHO based on vital registration data [17]. As presented in the flow chart of the study (Fig 1), for 8 surveys MMR cannot be reasonably determined using any of the aforementioned approaches, hence they were excluded from the analysis. In 31 surveys conducted in 1990’s, timing of the first PNC was inconsistently defined. Accordingly, proportion of mothers who had PNC in the first 2 days after birth was extrapolated from other DHSs conducted later in the same country. In 30 of the surveys, maternal thinness data were missing and the gap was filled using the Nutrition Landscape Information System (NLiS) of the WHO [18].

Regarding prevalence of anemia in pregnancy, in 111 surveys the information was available from the DHSs or it was interpolated from other DHS reports of the same country. For the remaining 137 surveys, country and year specific estimates were extracted from the World Bank database [19]. Prevalence of HIV among women of reproductive age was only measured in 61surveys. For the rest, data on the prevalence of HIV in the general population was taken from the World Bank database [20] and overinflated by a correction factor of 1.13 in order to accommodate for the higher prevalence of HIV among women of reproductive age. The inflation factor was determined by dividing the HIV prevalence among women reported by the DHSs for the 61 countries, by the prevalence in the general population estimated by the World Bank for the same set of countries.

According to the WHO, at population level C-section rates higher than 10% are not associated with reductions in maternal mortality; further, rates exceeding 15% indicate overutilization of the service [21]. Accordingly, in this analysis C-section rates above 15% were all trimmed to 15%.

### Model development and fitness assessment

Data were analyzed using SPSS software. Frequency distribution and measures of central tendency and dispersion were used to describe the data. Generalized Estimating Equation (GEE) with Negative Binomial probability distribution, log link function and unstructured correlation matrix was used to model MMR as a function of the covariates. Name of the country was set as ‘subject variable’ and the year the surveys were conducted was considered as ‘within subject variable’.

In the analysis, GEE was preferred over Mixed effects model because the main interest of the undertaking was to determine marginal (population average) changes. Further, the Negative Binomial GEE model was selected because it yielded smaller Quasi Likelihood under Independence Model Criterion (QIC), than the other two models (Poisson log linear model and Tweedie model with log or identity link) that can accommodate count response variable. Log-transformed linear model was not chosen because it provided negative predicted values for some of the observations.

Initially bivariable analyses were done for each of the predictor and all the significant covariates were considered as candidate variables for the multivariable model. The final model was built through a series of procedures involving removal of a variable with highest non-significant *p*-value at each step until all significant variables that best describe the response variable remained in the model. Stepwise regression was chosen so that predictor variables can remain as few as possible. Two or more alternative models were compared using QIC. In the multivariable models, the extent of multicollinearity among the covariates was determined using Variance Inflation Factor (VIF) and found to be within the acceptable limit.

The predicted and observed MMR values were regressed using simple least squares approach and the coefficient of determination (r-squared value) was used as a measure of proportion of variability explained by the model. Relative standard error (RSE)–standard error expressed as a percent of the estimate–was used to measure the precision of the model estimates.

With the intension of improving model fitness, sensitivity analysis was made by replacing modern CPR with three related variables (CPR for any contraceptive method, GFR and total fertility rate (TFR)) one at a time. Further trimmed CS rate was replaced by non-trimmed CS rate. But all measures did not improve the fitness and in most cases inflated the QIC.

Further, stratified analysis based on six geographical regions of the WHO and developing separate models for (sub-Saharan Africa) SSA countries and non-SSA countries were attempted. However, the analyses did not provide evidence of better model fitness. The results of the analysis are not included in this manuscript because the sample size did not allow for sensible stratified analysis.

### Application of the model to describe maternal mortality in Ethiopia

This paper also applied the model for describing sub-national variations of MMR in Ethiopia between 2000 and 2016. Ethiopia has so far conducted four DHSs in 2000, 2005, 2011 and 2016, and all estimated MMR at national level. Initially, the observed and model estimated MMRs at national level for the four time-points (2000, 2005, 2011 and 2016) were compared. Then for each eleven regions of the country, MMR was estimated for the four time-points based on region-specific level of the predictors as reported in the DHS reports [22–25]. Proportion of maternal death reduction between 2000 and 1016 was also computed for each region.

Further, at national and sub-national (region) levels, the number of maternal deaths in 2016 were estimated. Initially the expected number of live births in each region was calculated based on its population size, proportion of women of reproductive age and GFR. The population size and number of women of reproductive age were projected from the 2007 national census of Ethiopia [26] and region-specific GFRs were determined by reanalyzing the Ethiopian DHS 2016 data. Finally numbers of maternal deaths were estimated for each region using the MMR and expected number of live births.

## Results

### Characteristics of the surveys included in the model building

A total of 248 national surveys conducted in 80 countries between 1990 and 2017 were included. The median number of surveys per country was 3 and ranged from 1 to 9. Most of the studies (93.1%) were standard DHSs while the remaining were continuous DHSs, National Family Health Surveys or specialized maternal mortality surveys.

Regional grouping based on the WHO classification indicates about half of the surveys (51.2%) were conducted in the sub-Saharan Africa region. Region of the Americas (15.7%), South-East Asia (10.9%), Eastern Mediterranean (9.3%), European (7.7%) and Western Pacific (5.2%) regions were also represented. More than one-third (37.5%) of the surveys were conducted between 2000 and 2009; whereas, 30.6 and 31.9% were carried in 1990–99 and 2010–17 periods, respectively. According to the Word Bank classification, 63.7% of the surveys were conducted in either low- or lower-middle income countries.

Among the 250 surveys included in the analysis, 152 (61.2%) measured the level of MMR. For the remaining surveys, MMR was determined based on various methods and external data sources including: the WHO’s estimates based on national civil registration systems data (24.0%), interpolation or extrapolation based on other DHS surveys of the same country (10.4%), reports of other national surveys (3.2%) and official national reports (1.2%) (Table 1).

**Table 1:** Summary of the surveys included in the development of the model, 1990–2017.

Characteristics of the surveys (n = 248)	Frequency	Percentage
Type of the survey		
Standard DHS	231	93.2
Continuous DHS	7	2.8
Others	10	4.0
Geographical region		
Sub-Saharan Africa	127	51.2
Americas	39	15.7
South-East Asia	27	10.9
Eastern Mediterranean	23	9.3
European	19	7.7
Western Pacific	13	5.2
Year the survey was conducted		
1990–99	76	30.6
2000–09	93	37.5
2010–17	79	31.9
Average national GDP (PPP) per capital of the countries over the preceding 5 years of the survey		
Low-income	26	10.5
Lower-middle income	132	53.2
Upper-middle income	90	36.3
Source of MMR data		
DHS	152	61.3
WHO estimates based on national civil registration data	59	23.9
Interpolated or extrapolated based on DHS reports	26	10.4
Reports of other national surveys	8	3.2
Official reports	3	1.2

### Description of the potential predictors of MMR

Table 2 presents range of values observed for MMR and nine potential predictors used in the model. The median observed MMR was 371 deaths per 100,000 LB and ranged from 25 to 1,243. Low MMR (< 100 deaths/100,000 LB) was observed in 16.1% of the data-points whereas extremely high values (≥ 1,000 deaths/100,000 LB) were reported in 2.6% of the cases.

Wide ranges of values have been observed for the predictors including modern CPR among married women (1.0–86.7%), coverage of having 4 or more ANC visits (5.0–96.0%), health facility delivery (3.5–99.4%) and PNC utilization within the first 48 hours after birth (1.0–97.4%). Across the surveys C-section rate ranged from 0.4 to 56.4%. In 16.5% of the surveys the rates exceeded 15.0% and accordingly they were trimmed to 15%.

**Table 2:** Summary of the variables used in the model development.

Variables (n = 248)	Frequency	Percentage	Median (min—max)	Mean (±Standard deviation)
Maternal mortality ratio/100,000 live births				
Less than 100	40	16.1	371 (25–1,243)	393 (**±**270)
100–399	91	36.7		
400–699	90	36.3		
700–999	18	7.3		
1,000 or above	9	3.6		
Modern CPR among married women of reproductive age				
Less than 25%	106	42.7	27.4% (1.0–86.7%)	29.1% (±19.5%)
25.0–49.9%	89	35.9		
50.0–74.9%	49	19.8		
75.0% or above	4	1.6		
% of women who had 4 or more ANC visits				
Less than 25%	38	15.3	56.1% (5.0–96.0%)	53.8% (±24.2%)
25.0–49.9%	63	25.4		
50.0–74.9%	91	36.7		
75.0% or above	56	22.6		
% of women who gave the recent birth in health facility				
Less than 25%	33	13.3	55.2% (3.5–99.4%)	51.6% (±27.6%)
25.0–49.9%	82	33.1		
50.0–74.9%	62	25.0		
75.0% or above	71	28.6		
% of women who had PNC in the first 2 days after birth				
Less than 25%	113	45.6	30.7% (1.0–97.4%)	31.9% (±28.9%)
25.0–49.9%	52	21.0		
50.0–74.9%	50	20.2		
75.0% or above	33	13.3		
% of women who delivered the recent birth via C-section				
Less than 5%	123	49.6	5.0% (0.4–56.4%)	8.2% (±8.9%)
5.0–9.9%	61	24.6		
10–14.9%	23	9.3		
15.0% or above	41	16.5		
Prevalence of maternal thinness				
Less than 5%	54	21.8	9.8% (0.2–55.0%)	14.8% (±11.2%)
5.0–9.9%	72	29.0		
10–14.9%	49	19.8		
15.0% or above	73	29.4		
Prevalence of maternal short stature (n = 234)				
Less than 5%	166	71.6	2% (0.1–31.8%)	4.4% (±5.3%)
5.0–9.9%	31	13.4		
10–14.9%	22	9.5		
15.0% or above	13	5.6		
Public health significance of anemia				
None (0–4.9%)	0	0.0	46.9% (11.2–78.2%)	46.9% (14.2%)
Mild (5–19.9%)	6	2.4		
Moderate (20.0–39.9%)	79	31.9		
Severe (40% or above)	163	65.6		
Prevalence of HIV among women of reproductive age				
Less than 1%	127	51.2	0.9% (0.0–31.1%)	3.6% (5.5%)
1.0–4.9%	68	27.4		
5.0–14.9%	35	14.1		
15.0% or more	18	7.3		

Regarding maternal nutritional status, the prevalence of thinness among women of reproductive age ranged from 0.2 to 55.0%; whereas magnitude of short stature varied from 0.1 to 31.8%.The prevalence anaemia in pregnancy was from 11.2 to 78.2%. In the vast majority (97.5%) of the data-points the prevalence exceeded the 20% cutoff indicative of moderate or severe public health significance of anaemia.

The HIV prevalence rate among women of reproductive age varied between 0.0 to 31.1% and in 21.4% of the data-points the prevalence was at or above 5% representing generalized HIV epidemics (Table 2).

### Description of the model

Table 3 summarizes the findings of the bivariable analyses, and the regression coefficients for the final model (model 4) and three other intermediate models (model 1–3) along with their QIC. In the bivariaiable analyses all the nine predictors showed strong association with MMR (*P*< 0.001). As one may expect, higher magnitudes of CPR and better coverage of ANC, delivery in a health facility, PNC and C-section were all negatively associated with MMR. Further, MMR tends to rise with increasing prevalence of HIV, thinness and anemia. Nevertheless, unexpected negative association was observed between MMR and prevalence of maternal short stature.

**Table 3:** Summary of the predictors of MMR.

Predictors	Bivariable model		Multivariable models							
			Model 1^a^		Model 2^b^		Model 3^c^		Model 4^d^	
	*β*	*P*-value	*β*	*P*-value	*β*	*P*-value	*β*	*P*-value	*β*	*P*-value
Intercept	-	-	6.442	< 0.001	6.431	< 0.001	6.519	< 0.001	6.464	< 0.001
CPR^1^	-0.019	< 0.001	-0.013	< 0.001	-0.013	< 0.001	-0.012	< 0.001	-0.013	< 0.001
4 or more ANC visits coverage^2^	-0.012	< 0.001	0.004	0.180	0.004	0.179	-	-	-	-
Delivery in health intuition^3^	-0.016	< 0.001	-0.009	0.003	-0.009	0.003	-0.007	0.003	-0.006	0.004
Prevalence of C-section^4^	-0.100	< 0.001	-0.026	0.041	-0.026	0.037	-0.023	0.057	-0.027	0.017
PNC coverage^5^	-0.012	< 0.001	-0.004	0.006	-0.004	0.006	-0.003	0.033	-0.003	0.029
Prevalence of HIV^6^	0.046	< 0.001	0.057	< 0.001	0.057	< 0.001	0.059	< 0.001	0.060	< 0.001
Prevalence of thinness^7^	0.028	< 0.001	0.013	0.001	0.013	0.001	0.011	0.004	0.011	0.007
Prevalence of short stature^8^	-0.027	< 0.001	-0.011	0.064	-0.011	0.064	-0.009	0.108	-	-
Prevalence of anemia^9^	0.014	< 0.001	0.000	0.955	-	-	-	-	-	-
QIC	-	-	73.78		71.77		70.38		68.56	

^1^ Modern contraceptives prevalence rate among married women of reproductive age

^2^% of women who had 4 or more ANC visits during the recent pregnancy in the past 5 years

^3^% of women who gave the recent birth in the last 5 years in a health facility

^4^% of women who gave the recent birth via C-section

^5^% of women who had PNC in the first 2 days after the recent birth in the last 5 years

^6^ Prevalence of HIV among women of reproductive age

^7^Prevalence of thinness (BMI < 18.5 kg/m^2^) among women of reproductive age

^8^ Prevalence of short stature (height < 145 cm) among women of reproductive age

^9^ Prevalence of anemia among pregnant women

^a^ All the 9 variables included in the model

^b^ All the variables excluding prevalence of anemia included in the model

^c^ All the variables excluding prevalence of anemia and short stature included in the model

^d^ Final model comprising CPR, ANC coverage, Utilization of delivery service, CS rate, PNC coverage, Prevalence of HIV and Prevalence of thinness

The final model was built through a series of steps involving removal of a variable with highest non-significant *p*-value at each step until all significant variables remained in the model. In the first model all the 9 predictors were fitted into one multivariable Negative Log-binomial model. Then, after dropping prevalence of anaemia that had the highest non-significant *p*-value (*p* = 0.955), the second model was developed based on 8 variables. In the third and fourth models ANC coverage (*p* = 0.179) and prevalence of short stature (*p* = 0.108) were excluded consecutively. The ultimate model (model 4) comprised six significant variables: CPR, ANC coverage, delivery in health institutions, utilization of PNC in the first 48 hours, C-section rate and, prevalence of thinness and HIV.

Alternatively the final model can be rewritten as follows.

Ln(MMR)=6.464–0.013(CPR)–0.006(HID)–0.003(PNC)–0.027(CSrate)+0.060(HIVprevalence)+0.011(thinnessprevalence)

The coefficients of the final model can be interpreted in the following way. A percent increase in the coverage of CPR is associated 0.013 decline in MMR on the logarithmic scale. Similarly, one percent increase in the health institution delivery, utilization of PNC and C-section rate result in 0.006, 0.003 and 0.027 reduction in MMR on the same scale. Conversely, a unit increase in the prevalence of HIV and thinness results in 0.060 and 0.011 rise in MMR on the logarithmic scale.

### Model fitness assessment

**Fig 3:**
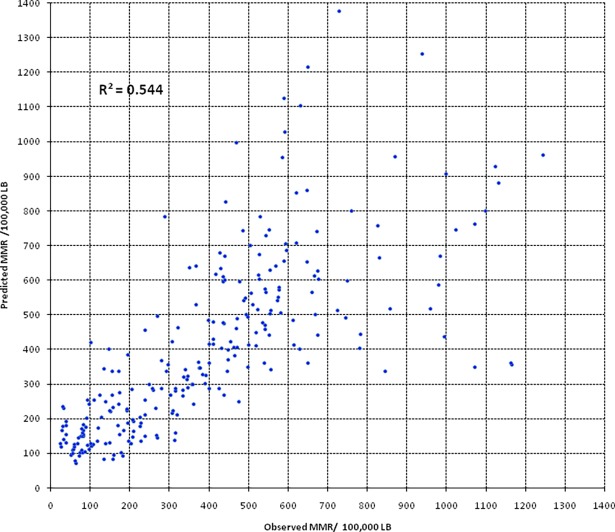
Relationship between observed and predicted values of MMR.

The strength of association between the observed and predicted MMR was assessed using simple linear regression analysis and the r-squared value was used as a goodness-of-fit measure. The r-square value was 54.3%. Fig 1 shows the relationship between observed and predicted values of MMR. Though linear association was evident, the plots get more and more dispersed from the perfect-fit line of Y = X with increasing observed MMR indicating the predicting ability of the model declines with increasing level of MMR. Simple linear regression analysis based on observed values of MMR less than 650 yields a better r-squared value of 64.3% (Fig 3).

**Fig 4:**
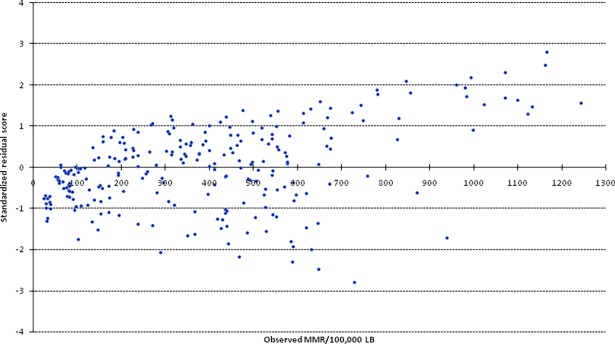
Standardized residual scores across different values of observed MMR.

The plots of the observed MMR versus standardized residual scores also suggested that model prediction errors tend to increase with increasing level of observed MMR. Especially when the magnitude of MMR in the study population exceeds 650 per 100,000 LB, the model systematically underestimates the true MMR (Fig 4).

### Precision of the model estimates

Relative standard error (RSE) was used to measure the precision of the model. The mean (±SD) RSE of the model estimates was 8.6 (±2.6) % and ranged from 4.1 to 19.8%. In 116 of the DHSs MMRs were reported with their confidence intervals, hence calculation of the RSE was possible. In the surveys the mean (±SD) RSE was 14.4 (±4.7) % and ranged 6.3 to 33.2%. The analysis indicates the precision of model-based estimates is better than survey-based estimates.

### Application of the model to describe the sub-national variation of MMR in Ethiopia

**Fig 5:**
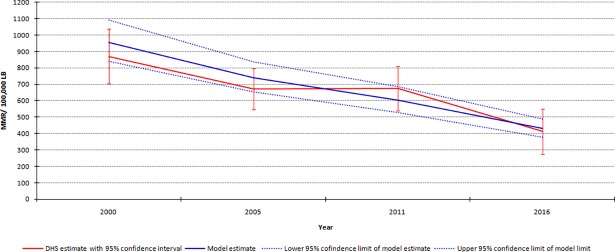
MMR in Ethiopia between 2000–1016 based on DHSs and the model estimates.

Ethiopia has so far conducted four DHSs and all the surveys estimated MMR at the national level. Fig 4 shows MMRs reported by DHSs and estimated by the model with their respective 95% confidence intervals (CIs) for the years 2000, 2005, 2011 and 2016. The point estimators of the MMRs determined by the DHSs all fall within the 95% confidence band of the model estimates indicating that the model has reasonably descried the maternal mortality pattern in Ethiopia. Further the precision of the model estimates–as measured by width of the confidence band–is better than survey-based estimates (Fig 5).

Sub-national model-based estimates indicated, in 2016 the highest MMRs per 100,000 LB were in Somali (805) and Afar (795) regions. Conversely, the lowest was in Addis Ababa city (129) and Harari region (329). In all of the regions, substancially decline in MMR was observed between 2000 and 2016. However, the rates of decline substancially vary across the regions. Somali and Afar regions only managed to reduce MMR by 26 and 30% over the period, respectively. On the other hand, in Addis Ababa city MMR was reduced by 73%.

The total number of maternal deaths in 2016 in each region was estimated based on the expected number of live births and model estimated MMR of the regions. According to the estimate, 16,083 maternal deaths occurred in Ethiopia in 2016. Nearly half of the deaths (45.6%)–equivalent to 7,326 deaths–occurred in Oromiya region alone. Other region-specific estimates are presented in the following table (Table 4).

**Table 4:** Estimated MMR and number of maternal deaths in 11 regions of Ethiopia.

Region	Estimated MMR per 100,000 LB (95% Confidence interval)				% reduction in MMR between 2000–16	Population size (2016)	Maternal deaths in 2016	
	2000	2005	2010	2016			Number	%
Addis Ababa	483 (404–577)	212 (175–256)	151 (114–201)	129 (97–171)	73.3	3,646,504	89	0.6
Affar	1,132 (920–1,393)	988 (858–1,137)	928 (766–1,123)	795 (684–924)	29.8	1,895,770	608	3.8
Amhara	923 (819–1,041)	750 (659–853)	594 (510–691)	369 (312–437)	60.0	22,128,094	2,058	12.8
Benishangul-Gumuz	783 (663–925)	816 (716–931)	616 (539–703)	471 (417–532)	39.8	966,787	158	1.0
Dire Dawa	705 (602–826)	527 (476–584)	455 (412–503)	372 (337–411)	47.2	472,892	41	0.3
Gambela	1,149 (907–1,457)	939 (799–1,104)	599 (499–719)	545 (462–642)	52.6	486,685	82	0.5
Harari	766 (674–871)	512 (469–558)	458 (408–513)	329 (298–364)	57.0	255,132	30	0.2
Oromiya	902 (803–1,013)	766 (675–869)	626 (547–717)	520 (459–588)	42.4	38,798,587	7,326	45.6
SNNPR	699 (790–790)	701 (419–794)	568 (491–657)	363 (307–428)	48.1	21,489,757	2,382	14.8
Somali	1,087 (861–1,371)	920 (795–1,065)	889 (779–1,015)	805 (712–911)	25.9	6,177,290	2,645	16.4
Tigray	901 (766–1,059)	848 (728–987)	717 (607–848)	374 (311–449)	58.5	5,951,317	664	4.1
**National**	957 (840–1,092)	741 (655–838)	603 (528–688)	430 (378–489)	55.1	102,403,196	16,083	100.0

## Discussion

The analysis suggested, in low- and middle-income countries where there is paucity of reliable and accurate maternal mortality data, MMR can be estimated with reasonable validity and precision based on six health-related indicators: CPR, coverage of health institution delivery and PNC, access to C-section, magnitude of thinness and prevalence of HIV. As sub-national information about the indicators is readily available through national surveys including the DHS and MICS, MMR can easily be estimated at sub-county level using the model.

The proposed statistical model comprising six predictors is compatible with the McCarthy–Maine model briefly described earlier in the methods section [16]. CRP can be categorized as a factor that reduces the likelihood that a woman will become pregnant; thinness and HIV status can be considered as attributes that affect the probability that a pregnant woman will experience life threatening pregnancy related complications; and, utilization of health institution delivery, PNC and C-section services can be taken as factors that improve the outcomes for women with complications [16].

Simple linear regression analysis indicated model predicted values explained about 55% of the variation of the observed MMR. Further, when the observed values are below 650 deaths/100,000 LB, the coefficient of determination increases to 65%. The r-squared value of the model was judged to be acceptable considering the fact that the observed MMRs themselves are not usually measured with high precision. Usually, due to rareness of maternal death in statistical context, MMR estimated though surveys suffer from high standard errors. A study indicated maternal mortality estimation based on routine sample surveys provides wide CI exceeding ± 15% [27]. Consequently, such uncertainties are likely to be carried forward and reduce the validity and precision of the model.

As described earlier, the model prediction errors tend to rise with increase level of MMR.

This can be due to the exclusion of variables like abortion and malaria in the model which have substantial contribution to the burden of maternal mortality in the developing world. According to a systematic review, in the developing regions 8% of all the maternal deaths are attributable to abortion [28]. Yet, the decline in the validity of the estimates especially when the observed MMR exceeds 650 deaths/100,000 LB might not seriously limit the applicability of the model because, according to the recent WHO estimate only 10 countries currently have national MMR exceeding the aforementioned level [4].

The mean ± SD of RSE of the model estimates was (8.6±2.6)% found to be lower than what had been reported in 116 DHS reports (14.4±4.7)%. This indicates the model-based estimates have better precision than MMR observed by DHSs. As described earlier, due to rareness of maternal death, survey based estimates tend to produce MMR with lower precision.

The analysis indicated increases in CS rate, CPR, health institution delivery and PNC are associated with significant decline in maternal mortality. All the observed associations are compatible with the theoretical understanding that access to family planning, and basic and emergency obstetric services are vital for reducing maternal death. According to a study, in 2008 worldwide contraceptive use averted nearly 272,000 maternal deaths and without contraceptives the estimated deaths would have been nearly doubled [29]. A mathematical model suggested, most low income countries have C-section rate below the WHO recommended level of 10–15% and increasing the rates to this threshold reduces maternal death by 60% [30]. A study estimated 16–33% of all maternal deaths can be averted through prevention and management of obstructed labour, pregnancy inducted hypertension, puerperal sepsis and obstetric hemorrhage by skilled attendance at delivery [31]. Regarding PNC, a systematic review concluded more than 60% all maternal deaths occur in the postpartum period and 45% of postpartum deaths occurred within the first day of delivery. Accordingly, providing early PNC averts the substantial proportion of maternal deaths [32].

The analysis showed increase in HIV prevalence among women of reproductive age is associated with significant rise in MMR. This is consistent to the understanding that globally 6–20% of maternal deaths are attributable to HIV infection and HIV positive pregnant women have eight to ten-fold increased risk of dying during pregnancy compared with HIV negative women [33,34]. On the other hand, as high maternal mortality and HIV prevalence tend to co-occur in the developing world, the observed strength of association might have been overestimated.

The consequence of maternal thinness on fetal growth and undesirable birth outcomes is unequivocal [35]. However, there is limited evidence regarding its effect on maternal mortality. The current analysis indicated, at population level increase in the prevalence of maternal thinness is associated with significant rise in the magnitude of MMR. A prospective cohort study in US concluded low pre-pregnancy BMI, compared with normal BMI, is associated with a statistically significant rise in severe maternal morbidity and mortality [36]. The recent Lancet Maternal and Child Health Series concluded iron and calcium deficiencies which are likely to co-occur with maternal undernutrition, substancially increase risk of maternal deaths [37].

Studies suggested short maternal stature is an independent risk factor of obstructed labor and Cesarean delivery [38,39]. However in this analysis, no relationship was observed at population level between magnitude of maternal short stature and MMR. Even, a negative but non-significant relationship was witnessed. This can possibility be due to the influence of data from countries like Philippines, Peru, Guatemala that have relatively lower MMR but very high prevalence of maternal stunting. Conversely, countries like Senegal, Gambia, Burkina Faso, Chad and Niger have extremely low prevalence of maternal short stature but high MMR.

In the model building process it was observed that coverage of frequent ANC visits does not explain population level variability of MMR. Other studies have also suggested that ANC may fail to improve maternal outcomes due to multiple reasons including poor quality of service and difficulty in predicting birth complications ahead of time [40,41]. Further, in this specific analysis, the fact that ANC coverage was adjusted for utilization of delivery service and PNC can also underestimate its benefit because prenatal care may reduce maternal death through enhancing the utilization other maternity services.

Prevalence of maternal anemia also failed to be a significant predictor of MMR at population level. This is against the understanding that anemia is an important cause of maternal mortality [3,26]. A systematic review concluded a g/dl increase in population mean hemoglobin could reduce the risk of maternal mortality by 25% [42]. The unexpected finding might be due to multiple reasons. Firstly, in all of the surveys included, anemia had at least mild public significance and many central Asian counties including Azerbaijan, Tajikistan, Uzbekistan and Turkmenistan had high prevalence of anemia (>40%) despite having relatively lower MMR. This might have diluted the significance of anemia in the model. Secondly, due to lack of data, the analysis was limited to the total prevalence of anemia rather than severe anemia, which is considered to be a stronger and direct predictor of maternal mortality.

As compared to earlier models that endeavored to estimate MMR, the typical advantage of the current undertaking is that all the parameters are based on health-related indicators and reliable data is likely to be available for all of them at sub-national level. Further, the model enrolled additional covariates including C-section rate, utilization of PNC and prevalence of maternal thinness which have critical relevance to maternal deaths in the developing world. Though having several predictors within a model is not a desirable feature, the variables have improved its fitness and make it more sensible from clinical or public health perspective of view.

As described earlier, DHS typically determine PRMR over a typical reference period of 5 to 7 years based on sisterhood method and directly interpret it as MMR [14]. In the model development, the PRMR reported in the DHS was treated as MMR and no attempt was made to adjust for non-maternal deaths that can occur during pregnancy or in the postpartum period. This is because no information was provided in the DHS to differentiate accidental and incidental deaths during pregnancy from true maternal deaths. Hence the model estimates might have overestimated the level of MMR.

Further, the MMR estimation of the DHS suffers from multiple methodological shortcomings and these limitations are likely to affect the validity and accuracy of the model-based estimates. Though most DHS determine PRMR with a reference period of 5–7 years, some surveys use different timeframes. Yet, the model assumed the number of years covered by the estimate is consistent and this may compromise the precision of the estimates. One of the major challenges of determining MMR through surveys like the DHS is the problem with small number of maternal death. Due to the statistical rareness of the pregnancy related deaths, usually DHS determine PRMR with low precision and this may affect the precision of the model estimates too. In this study, RSE was provided for both observed and predicted values as a measure of precision so that readers can clearly understand the reliability of the model.

The application of the model should also be made in consideration of the following limitations. Firstly the validity of the model was only assessed based on the value of the coefficient of determination and more rigorous evaluation using external testing data has not been attempted because of the paucity of eligible surveys. Secondly, the model was developed based on the data of low- and middle income countries that typically have high MMR; as a result, its performance in predicting values below the observed range is questionable. Thirdly, despite the model considered a number of potential predictors, other essential variables like coverage of safe abortion care and prevalence of malaria have not been included. Further, the model only focused on coverage maternity services without considering their quality. This might have underestimated the contribution of the services for the reduction of maternal deaths. Finally, as the model was developed based on population level data, ecological fallacy is possible and extrapolation of the findings to individual level is unreasonable.

## Conclusion

The analysis suggested, in low- and middle-income countries where dependable maternal mortality data are not available, MMR can be estimated with reasonable accuracy and precision at national and sub-national levels based on six health indicators: CPR, coverage of health institution delivery and PNC, access to C-section, magnitude of thinness and prevalence of HIV.

## Supporting information

S1 Appendix Supporting data. (XLSX)
